# Clustering and Flow Conservation Monitoring Tool for Software Defined Networks

**DOI:** 10.3390/s18041079

**Published:** 2018-04-03

**Authors:** Jesús Antonio Puente Fernández, Luis Javier García Villalba, Tai-Hoon Kim

**Affiliations:** 1 Group of Analysis, Security and Systems (GASS), Department of Software Engineering and Artificial Intelligence (DISIA), Faculty of Computer Science and Engineering, Office 431, Universidad Complutense de Madrid (UCM), Calle Profesor José García Santesmases 9, Ciudad Universitaria, 28040 Madrid, Spain; jesusantoniopuente@ucm.es; 2 Department of Convergence Security, Sungshin Women’s University, 249-1 Dongseon-Dong 3-ga, Seoul 136-742, Korea; taihoonn@daum.net

**Keywords:** clustering, data plane, flow conservation, software defined networks, statistics, videostreaming

## Abstract

Prediction systems present some challenges on two fronts: the relation between video quality and observed session features and on the other hand, dynamics changes on the video quality. Software Defined Networks (SDN) is a new concept of network architecture that provides the separation of control plane (controller) and data plane (switches) in network devices. Due to the existence of the southbound interface, it is possible to deploy monitoring tools to obtain the network status and retrieve a statistics collection. Therefore, achieving the most accurate statistics depends on a strategy of monitoring and information requests of network devices. In this paper, we propose an enhanced algorithm for requesting statistics to measure the traffic flow in SDN networks. Such an algorithm is based on grouping network switches in clusters focusing on their number of ports to apply different monitoring techniques. Such grouping occurs by avoiding monitoring queries in network switches with common characteristics and then, by omitting redundant information. In this way, the present proposal decreases the number of monitoring queries to switches, improving the network traffic and preventing the switching overload. We have tested our optimization in a video streaming simulation using different types of videos. The experiments and comparison with traditional monitoring techniques demonstrate the feasibility of our proposal maintaining similar values decreasing the number of queries to the switches.

## 1. Introduction

Current internet subscription and business advertisement models for Internet video content are subject to achieving sufficient Quality of Experience (QoE) level in the video delivery. Moreover, the increase of video streaming content requires the provision of computation and infrastructure to satisfy these levels of QoE and High Quality (HQ). In the same way, High Definition (HD) content traffic has already overcame Standard Definition (SD) traffic, making HD video the most consumed by users [1]. Due to emerging online streaming video platforms as YouTube and Netflix, the HQ online video streaming has become an essential part of life for people around the world.

Most of the video service providers are always looking for the fastest delivery of their contents to clients in Content Delivery Networks (CDN). Nevertheless, the speed of this content depends on the network status and network conditions. Due to these conditions, the user (client) will experiment with better or worse quality of experience (QoE) receiving the video content. Then, if there is no resources provision and adaptation in the user applications, the traffic control could cause network congestion, frozen images or loss of coordination between audio and video during a video streaming. This problem has a direct impact on the degradation of video quality and experience on different user devices.

A new concept of network architecture such as Software Defined Networking (SDN), decouples the network control (control plane) from the underlying network resources (data plane) being controlled through a centralized controller using OpenFlow as standard protocol in their communications. Moreover, it also provides a set of application programming interfaces (APIs) to abbreviate the implementation of network services as routing, QoS, QoE, access control, and load balancer among others. In addition, it provides per flow statistics collection primitives to be queried at any time. Therefore, using this global view of the network is an essential advantage to developing sophisticated and complex monitoring tools. Taking into account these benefits, general lines of a well-designed network monitoring framework are a broad selection of network metrics as data rate, error rate among others to monitor different levels of aggregation.

The appropriate SDN-based network behaviour depends on the capacity of the controller to make good decisions. The controller can not only correct failures inside the network, but it can also prevent future issues based on the available monitoring information. For instance, the controller can prevent DDoS or a decreasing of QoS/QoE analyzing metrics as data rate, packet loss or delay in the links within the network. Therefore, introducing SDN enables the exchange of network information with flexibility and adaptability.

To improve video QoE, it is important to obtain in every moment a full and accurate state of network resources. These data are very useful to build prediction systems or monitoring tools [2] that are able to suggest the best path for data delivery, especially video content. However, applying monitoring tools in networks usually requires the installation of costly hardware and software. A solution for providing monitoring metrics to the SDN controller is the analysis of the information provided by the OpenFlow Protocol. However, the use of the OpenFlow Protocol to request switch information increases the load of data and control planes [3]. If the controller continuously requests information to every switch in the network, it provides accuracy in the monitoring information, but this process generates an additional task in network switches and therefore, increasing the load of the controller affects other demanding tasks. Then, the optimization of controller requests is an open challenge in the research community. In the present work, we analyze the most relevant contributions in this topic.

In addition, we present a new enhanced controller that decreases the number of monitoring queries in the switches of the topology without compromising the accuracy of monitored values. For this reason, we reduce the request of the switches that do not offer valuable information for the analysis. This controller enhancement consists of two techniques: the first choice employs the flow-conservation algorithm for switches that are composed of two ports and the second option uses supervised learning as clustering. Such flow-conservation law states that all incoming data to the switch will be the same that it forwards to the remaining port. Then, the value of the outgoing link of a switch can be estimated with the value of the incoming link reducing to half the monitoring request in such cases. The other enhancement is grouping the network switches in clusters to apply random queries to them instead of querying all of them. In both cases, remaining values of non-monitored switches will be calculated with the information from neighbouring switches using the principle of conservation of currents flows.

To demonstrate the feasibility of our monitoring tool, we use a network emulator to build a testing scenario in which the two monitoring techniques proposed in this paper are tested in a video streaming delivery between two hosts (client and server). Such simulations have been separated in two study cases: Study Case A and Study Case B. Study Case A has been focused on testing the effectiveness in the reduction of monitoring queries comparing statistics of data and error rate in two links of the topology. Study Case B has been performed to further check the results of the enhanced algorithm with other types of videos.

The rest of the paper is outlined as follows: Section 2 explains the Software Defined Networks and their architecture focusing on the OpenFlow Protocol and its packet processing which is the most extended in SDN. Related works are described in Section 3. The proposed algorithm to enhance the monitoring request is explained in Section 4. To check the well behaviour of the present algorithms, Section 5 contains the simulations and results of the testing. Finally, Section 6 concludes with a short discussion and conclusions.

## 2. Software Defined Networking and OpenFlow Protocol

In this section, we provide a general overview of Software Defined Networking and the protocol that it utilizes in its communication.

### 2.1. Software Defined Networking

**Figure 1:**
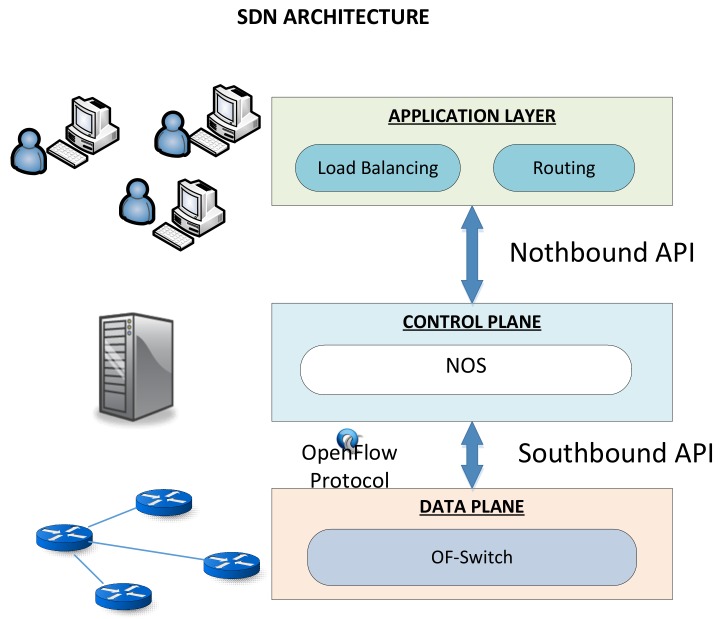
SDN Architecture.

Software Defined Networking (SDN) is a new network paradigm that gathers the key features from traditional networks: centralized network management, active networks and network virtualization. SDN was created to facilitate innovation since it enables simple programmatic control of the network behaviour: traffic data-path, load balancing among others. SDN facilities network management by decoupling the control plane (the decisions within the network) from the data plane (underlying network devices) as Figure 1 depicts. In this way, it removes the rigidity of static protocols since both planes are placed together.

Inside, the controller is running a high-level software application that provides a fast network management. Therefore, this centralized controller does not require individual configuration of network devices since every network behaviour is managed through the controller. Applicability in fields such as home networking, research campus, data centers, Internet of Things (IoT) among others [4] is carried out by SDN and managed by the OpenFlow [5,6] protocol. This protocol is the most extended in SDN and its maintenance and specification are the responsibility of the Open Networking Foundation-ONF [7]. In the next section, we provide a short overview of the OpenFlow protocol.

Logically, SDN is divided into three layers: data layer, control layer and application layer [3,8,9]. Network resources (data layer) are connected to the control layer through southbound interfaces. These interfaces provide the abstraction of the programmable switches and the connection with the software that is running within the controller. OpenFlow is the most representative example of this type of interfaces. As mentioned before, over these interfaces is running a Network Operating System (NOS) which is responsible for controlling network behaviour. Different NOS are available attending to the programming language that are been developed: NOX/POX [10], Maestro [11], Beacon [12] Floodlight [13], ONOS [14] and OpenDaylight [15]. Continuing with the logical design, the control plane is connected to the application layer through northbound interfaces which allow the creation of both applications and high-level network policies that are sent to the NOS. In the same way, examples of northbound interfaces are Frenetic [16,17], Procera [18], Netcore [19] and McNettle [20].

### 2.2. OpenFlow Protocol

OpenFlow [5] is the most extended used protocol in SDN. Its best feature is that the research community can contribute to its development and implementation of a wide range of functionality since it is open source. It defines the communication between the Layer 2 network devices (switches) and the controller. In a deeper view, it provides the ability to program the flow tables placed within a switch and it is also able to change or introduce new functionality at run-time.

The OpenFlow architecture [5] follows the SDN principle of decoupling data and control planes. It is based on three main entities: an OpenFlow switch (data plane), an external controller (control plane) and the OpenFlow Protocol [5]. This protocol is in charge of the communication between the controller (control plane) and network switches (data plane) through a secure channel. For more information, the OpenFlow Switch Specification [6] describes the requirements of an OpenFlow Switch.

## 3. Related Works

Monitoring tools are used to retrieve a global view of the traffic that is in the network. Due to the existence of multiple vendors, several monitoring tools are proposed. Such tools are very useful for network administrators to analyse the network for preventing future congestion problems, illegitimate traffic flows among others.

Monitoring networks is a task that can be performed taking into account two approaches: passive and active methods. Each one has their advantages and disadvantages but both values should be complementary. Moreover, we distinguish between SDN oriented and non-SDN oriented works.

Due to the recent spreading of Internet video delivery, it is expected to be deployed more broadly over the next few years. In this way, one of the key video enhancing solutions based on a onSDN oriented approach is HTTP adaptive streaming (HAS) [21]. As a relatively new technology in comparison with traditional push-based adaptive streaming techniques, deployment of HAS presents new challenges and opportunities for content developers, service providers, network operators and device manufacturers.

This is not only via traditional computers. Works like Amram et al. [22] propose a novel dynamic transport architecture for next generation mobile networks adapted to video service requirements. Its main novelty is the transport optimization of video delivery that is achieved through a QoE oriented redesign of networking mechanisms as well as the integration of CDN techniques.

Passive methods are helped by special purpose hardware as sniffers or built into other devices such as routers, switches or hosts. Their main characteristic is that they do not increase the traffic within the network when they are measuring statistics. This approach is highly valuable in network trouble-shooting but limited in isolating the exact fault location. Due to all packets inspections by the passive approach, security related issues are generated about how the data gathered from the network is protected.

Active methods are focused on the injection of packets within the network sending packets to network devices as switches and servers among others. Contrary to passive methods, active methods have a direct impact on network traffic since they increase such traffic. Such packets added to the traffic are called probe packets and their main function is to obtain and to measure statistics as delay, round-trip time among others.

Protocols such as Network Management Protocol (SNMP) [23] and Network Configuration Protocol (NETCONF) [24] allow for the monitoring of network devices and statistics using the passive method in traditional networks. In the same way, monitoring tools to estimate both sample and complete traffic statistics in flow based networks are NetFlow [25] and sFlow [26].

Continuing with monitoring tools, jFlow [27] is an extension of Java Language [28] that allows checking flow annotations in a static way. Moreover, it provides a compiler that checks (also statically) and prevents information fugues via storage channels. jFlow offers features that make flow checking less restrictive than other languages. These features are: a decentralized label model which enables the creation of privacy policies, an access control that permits code privileges and change them, a label polymorphism in the data classes, a run-time label checking to ensure that the information is not leaked and finally automatic label inference to abbreviate annotations that in other cases are required. Then, taking advantage of all these features, jFlow is a powerful tool to obtain flow statistics across the network.

Regarding SDN oriented approaches, one of the main proposals related with network monitoring is SuVMF [29] that provides a novel architecture for SDN large-scale networks based on a Software-Defined Unified Virtual Monitoring Function. This work also uses a passive method to monitor and it consists of three important entities that are responsible for monitoring management, intelligent control and modules that filter and transform the data. The statistics collection utilizes several passive methods such as sFlow [26] and SNMP [23] among others.

Sandor et al. [30] propose a three-tier architecture that implements response and reconfiguration capabilities in an industrial control system. It adopts a SDN tier for dynamic communications flow (re)configuration and whitelisting, an application tier for the optimal placement of anomaly detection systems and a supervision tier for gluing the three tiers together. Continuing this line, Genge et al. [31] propose a novel SDN controller named OptimalFlow that redesigns the network according to the solutions delivered by an integer linear programming (ILP) optimization problem. Such ILP encapsulates a shortest path routing objective and harmonizes ICS flow requirements including quality of service, security of communications, and reliability. Moreover, it also exposes two communication interfaces to enable a hierarchical control plane. Its northbound interface reduces a complete switch infrastructure to an emulated (software) switch, while its southbound interface connects to an OpenFlow controller to enable the monitoring and control of realemulated switches.

In [32], the authors use passive and active methods to measure network performance. It uses beacons to send probe packets and install additional flows in switches. Then, these beacons are used to estimate the packet lost rate and delay. A hybrid solution between passive and active methods is the framework besought in [33]. This work proposes a network-monitoring framework based on an orchestrator module with a flexible method to retrieve network statistics. Moreover, it creates a user profile based on its needs, retrieving statistics with passive (data rate and error rate) and active (probe packets to the switches) methods.

Works like OpenNetMon [34] monitor per-flow metrics in OpenFlow networks. It is specialized in throughput, delay and packet loss to determine if the end-to-end Quality of Service parameters are met to find suitable paths. It looks for the minimum number of queries to switches in order to obtain the metrics. The time interval of these polls depends on the increase-decrease of its past values. Applying different strategies to query for statistics can help to reduce the overhead in switches as well as in networks. In this order, OpenTM [35] proposes to follow a non-uniform querying distribution respect uniform schemes. This shows that this strategy is much faster than the existing ways of traffic estimation in IP networks.

Chowdhury et al. [36] propose Payless, a lowcost efficient network monitoring framework for SDN. Its main feature is that it provides an abstract view of the network and a regular manner to request statistics about the network resources. Moreover, since it has been developed as a set of connectable components, it provides interfaces to connect all of them (highlevel RESTful API). Then, a developer is able to insert custom components in the Payless framework and therefore, change its behaviour. Payless is based on the following components: a Request Interpreter that is responsible for transferring high level primitives to flow level primitives. In addition, it contains a Scheduler that polls the switches in order to collect per flow statistics, per flow aggregate statistics and per queue statistics. To detect the set of switches that have to be queried in a scheduled statistics collection, the Switch Selector component identifies them based on parameters (flow, aggregate or queue). Finally, an Aggregator & Data Store module is liable for collecting raw data from selected switches by the Switch Selector and also for storing such data in the data store which is an abstraction of a persistent storage system.

Continuing with monitoring in flow networks, Flowsense [37] is an approach that looks for high accuracy statistics with zero measurement cost using the physical separation of the control and data planes in SDN. It takes into account three properties: the duration that the flow takes in the entry of the flow table, the quantity of traffic that matches with that flow and finally, the input port of traffic that matches the entry. Nevertheless, Flowsense has some limitations: it seriously depends on the type of traffic that it is going to monitor since large flows as a video transmission can delay its computation and utilization. In addition, it is limited when it is trying to capture instant usage in any moment of the monitoring process.

Yu et al. [38] propose OpenSketch, a traffic measurement scheme for SDN that separates the measurement data plane from the control plane. For the control plane, OpenSketch provides a library that is responsible for configuring the pipeline and allocating the network resources for multiple measurement labours. For its part, it provides in the data plane a three-phase pipeline: hashing, filtering and counting. The first stage collects the packets source field and applies a hashing function to them. After that, the filtering stage gathers the packets destination field and filters them according a matching rule. Each matching rule has assigned an index field that will be used in the third phase to calculate the counter location. These steps intend to reduce the switches memory and maximize the accuracy in the measured values of the monitored network.

Continuing in this line, FlowCover [39] is also a low-cost scheme for monitoring SDN that collects the statistics of the network. It considerably reduces the communication traffic due to the aggregation request and replies against leaving the global view of the network. Moreover, it takes into account the real traffic across the network changing the polling scheme of the request. The architecture of FlowCover consists of three layers: OpenFlow Network Layer, FlowCover Core Layer and Monitoring Applications Layer. The OpenFlow Network Layer is composed of the network devices and the connections between them and the controller. For its part, the FlowCover Core Layer is the main component of the framework. The switches send the arrive/expire messages to the Core Layer and then, it forwards the messages to the routing module and flow state tracker. While the path is being calculated through the routing module, the state tracker holds the active flows in the network in real time. Therefore, the monitoring scheme takes all this information and calculates an effective polling plan and forwards it to the flow state collector. The last layer, Monitoring Applications Layer, is a set of multiple monitoring tasks such as link utilization, traffic matrix estimation, among others.

Another strategy to take into account is duplicating the traffic and sending it to a monitoring agent. This idea is used in MonSamp [40] where the authors create two agents named as collector and analyser to continuously read the flows within the switches. The algorithm will increase or decrease the flow rules in switches based on the capacity of the monitor and the network links congestion. The properties of these monitoring strategies can be used in parallel with other modules to get better results in terms of QoS among others. In [41], a framework for optimized multimedia routing in combination with a monitoring module is presented to provide QoS in different multimedia services. Its best advantage is the easy way to add routing algorithms to get the best path in the data sending. In the same way, Georgopoulos et al. [42] propose an OpenFlow-assisted QoE Fairness Framework that aims to fairly maximize the QoE of multiple competing clients in a shared network environment. By leveraging a Software Defined Networking technology, such as OpenFlow, they provide a control plane that orchestrates this functionality.

## 4. Enhanced Monitored Algorithm

The high performance of video content and other applications is a consequence of the network state and network components. In other words, if the network load is not so high, the controller will balance its resources to the application or service that demands such resources. Because of this, monitoring tools are important in networks with big demands of computation. To get the best network performance and response, it is important not to load the CPU and memory of the controller with statistics queries messages. In this way, we analyze the network topology and look for strategies to enhance these statistics queries.

### 4.1. Network Notation

Our network abstract model is gathered in Table 1. The network topology is represented as a directed graph G=(S,L) where $S=\{s_{1},s_{2},\ldots ,s_{n}$} denotes the set of switches and $L=\{l_{1},l_{2},\ldots ,l_{nl}$} represents the set of links between switches. Additionally, the set of clusters is denoted as $C\;=\{c_{0},c_{1},\ldots ,c_{nc-1}$} where each cluster is at the same time a set of switches. For instance, the arc(i,j) represents the link from source switch *i* to destination switch *j*. We let ns=|S|, nl=|L| and nc=|C| denote the number of switches, links and clusters in the topology respectively. Also, we define degree of a switch (number of incident links) like $deg(s_{i})$ where $s_{i}\in S$. We assume that ns,nl and nc are finite and $deg(s_{i})>0$.

In addition, we assume that the monitored data is grouped in packet flows *F*. Thus, all data packets that are sent in *G* belongs at least, to one packet flow $F_{i}\in F$. Each $F_{i}$ has a path from its source switch $s_{i}$ to its destination switch $s_{j}$.

### 4.2. Flow-Conservation Algorithm Enhancement

One of the enhancements used in this paper is the framework for monitoring SDN networks proposed in [43]. This framework divides the control layer in several modules for performing different operations and provides network statistics to the Application layer. In this way, the technique performed by these modules reduces the number of statistics queries that the controller performs to the switches of the topology. Such a reduction is due to the utilization of the flow conservation algorithm in 2-grade switches since all data traffic that the switch incoming port receives will be forwarded to the outgoing port of the same switch and therefore, the traffic over such links will be nearly the same.

### 4.3. Clustering Enhancement Technique

The other monitoring enhancement technique proposed in this paper is based on clustering methods. The term of clustering corresponds to a procedure that groups a set of objects or various subsets in a way that the objects share one or more characteristics in common. In our case, the common feature between all switches of *S* will be their number of ports and their critical value.

Applying clustering methods in a set of objects to be studied offers several benefits in terms of performance, scalability and management among others. These benefits are traduced in this research work as follows: firstly, using clustering methods for monitoring statistics increases the performance in the SDN infrastructure due to the reduction of traffic flow to the SDN controller. Secondly, applying our clustering enhanced algorithm offers scalability since no matter the number of network devices (e.g., a huge SDN network with more than a single SDN controller) in the infrastructure. Finally, using clustering methods offers a simplification in the switches management since similar switches can be handled in the same way.

The decision of using the number of ports as a common characteristic of network switches has been reached due to the probability of the traffic density that is flowing within such switches. Besides, the number of ports of a switch implies directly in the probability of the possible number of paths to forward the incoming data within them. Therefore, the greater the number of ports, the greater the probability that the switch is being used in a data path due to the number of possible destination switches that is connected to itself.

It is important to clarify that as an initial step of this work, the authors have tried to find a solution for pure SDN network infrastructures.

Before starting to request statistics to switches, Clustering_Function(G) reads the network topology structured in a graph G=(S,L) that will be used to create the clusters. Since *G* contains the network graph, the function Create_Cluster(G,Algorithm,N) divides it in *N* clusters, defined as $c_{0},c_{1},\ldots ,c_{nc-1}$. This switches grouping is based on the number of ports that they are composed of, applying one of the available algorithms: K-Means, Hierarchical, Expectation-Maximization or Density Based. Next, a short explanation about the different clustering algorithms:

- K-Means: is a clustering method that aims to divide *n* observations into *c* clusters based on the nearest mean of each observation (single mean vector).
- Hierarchical: is a method of cluster analysis that builds a cluster hierarchy based on the distance of the objects’ connections.
- Expectation-Maximization (EM): is an iterative method to look for maximum likelihood or maximum. In other words, it estimates the means and standard deviations for each cluster to maximize the likelihood.
- Density Based: is a clustering algorithm that defines areas with higher object density than the rest of the data set.

Once the algorithm has been selected, the criteria to build the clusters are the following:

- Switches that contain two ports (incoming and outgoing) will be grouped in a cluster to which the flow conservation algorithm (proposed in [43]) is applied.
- In other cases, clusters that contain switches with three or more ports will be grouped by their criticality. This critical value is assigned to each switch depending on how many times such a switch appears in all available paths to send the data from the client host to the server host.

The clustering process described above is gathered in the Create_Cluster(G,Algorithm,N) function in Algorithm 1. Concretely, function Load_Instances() goes over all switches and assigns them values of how many ports they have and in the same way, function Calculate_Criticality() assigns them values of their criticality. Next, the grouping step is performed through the Run_Clusters(S,Algorihtm,N) function introducing one of the implemented clustering algorithms (K-Means, Hierarchical, Expectation-Maximization, Density Based) and the number of clusters nc as parameters.
**Algorithm 1:** Create Cluster Function. **Input:**  Network Graph G(S,L)  Algorithm Alg  Number of Clusters *N* **Result:**  Set of clusters *C* 
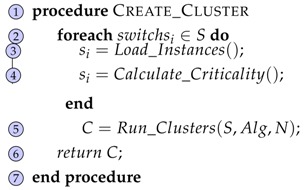


Once the clustering process has been performed, our monitoring tool starts to gather network information in each monitoring period $t_{mon}$. In order to reduce statistics queries, we apply different strategies in each network cluster:

- The cluster that contains switches with two ports ($deg(s_{i})=2$ for all $s_{i}\in c_{j}$) applies the enhancement based on the flow conservation algorithm proposed in [43] as Section 4.2. In this way, Algorithms 2–4 will be applied to cluster $c_{i}$ = $\left(S_{i},L_{i}\right)$ that will be set as the input graph of Algorithm 2. Therefore, these switches are released from being queried since their data will be obtained through their neighbouring switches.
- For clusters that contain switches with three or more ports, *N* switches (where *N* is a percentage of the total number of switches of each cluster fixed by the user) are randomly chosen in each $t_{mon}$ period. In this way, all switches of each cluster will be monitored but not in the same period.
- Another strategy that contains switches with three or more ports is based on choosing the most concurrent switches (in terms of traffic) in the same monitoring period. Hence, switches that are not forwarding traffic will not be monitored and the number of statistics queries will decrease.

Clusters are used by the controller to perform the queries as the Algorithm 2 states. For experimental proposes, we choose data rate and error rate as metrics to demonstrate the feasibility of our proposal.

Data rate is a monitoring metric that can prevent links congestion, links failures, DDoS attacks among others. Therefore, the controller starts the topology monitoring going over all clusters obtained in the Algorithm 2. The cluster that contains 2-grade switches will not be monitored since their values will be calculated with neighbour switches’ data rate values using Neighbour_Switch() function. In other cases, for clusters $c_{j}$ that contain switches whose grade is greater or equal to three, the controller chooses randomly *N* (value fixed) switches $s_{i}\in c_{j}$ using a passive method ofpt_port_stats(node,port(j)) provided by Floodlight [13] to send the request (OFPT_STATS_REQUEST) to each switch $s_{i}$ through a secure channel. Once the switch has received such a message, it responds to the controller with another message called (OFPT_STATS_REPLY) indicating its state. The controller receives such a response and identifies the counter of such a switch with its port in the topology. Sent data rate $d_{s_{ij}}$ is calculate as the difference between the bytes sent ($d_{s_{ij}^{k}}$−$d_{s_{ij}^{k-1}}$) in a monitoring period $t_{mon}$. To get $d_{s_{ij}}$ original metrics, the controller contrasts if the links of the switches are being monitored in such a period.
**Algorithm 2:** Calculate Data Rate Function. **Input:**  Set of Clusters *C* **Result:**  Data rate *d_s_ij__* sent for each *arc*(*i*, *j*) ∈ *L* 
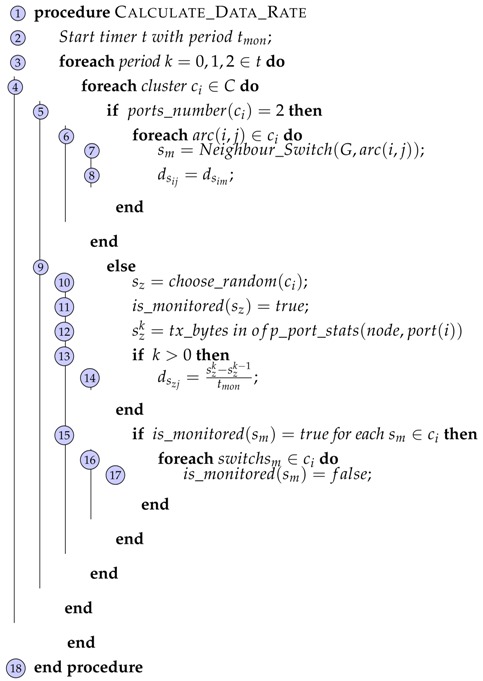


Therefore, applying these enhanced techniques, we are decreasing the number of switches requested with respect to a non-enhanced technique such as monitoring tools that monitor all switches of the topology.

Continuing with the network metrics, the error rate is the measure that calculates the packet loss percentage in a link. Algorithm 3 is the procedure that is responsible for calculating the error rate in an arc(i,j) within the topology.
**Algorithm 3:** Calculate Packet Loss Rate Function. **Input:**  Network link *arc*(*i*, *j*)  Monitoring period *t_mon_*  Time period *k* **Result:**  Packet loss rate *lr_ij_* for *arc*(*i*, *j*) 
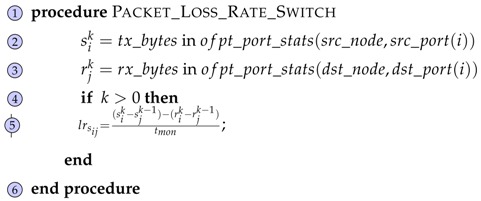

**Algorithm 4:** Calculate Error Rate Function. **Input:**  Set of Clusters *C* **Result:**  Error rate *l_r_s_ij___* for each *arc*(*i*, *j*) ∈ *L* 
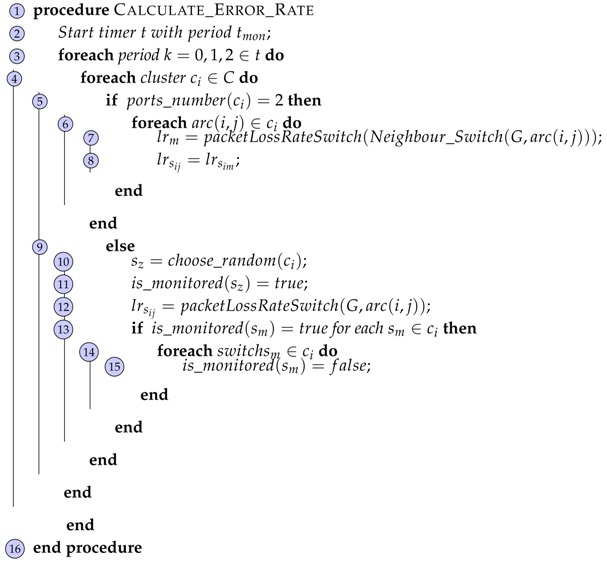


The controller uses a passive method ofpt_port_stats(node,port(i)) that sends port request (OFPT_STATS_REQUEST) to switches $s_{i}\in c_{j}$ through a secure channel. Once the switch receives this message, it replies its state to the controller with the (OFPT_STATS_REPLY) message. The controller receives the response and identifies the counter with the corresponding switch and port in the topology. Then, the error rate value in a link arc(i,j) defined as $lr_{s_{ij}}$ or $lr_{s_{ij}}^{\prime }$ is calculated as difference of the $d_{s_{ij}}$ or $d_{s_{ij}}^{\prime }$ sent bytes in the source switch and source port $s_{ij}$ or $s_{ij}^{\prime }$ minus the incoming bytes in the destination port of the destination switch $r_{s_{ij}}$ or $r_{s_{ij}^{\prime }}$ in a period $t_{mon}$.

### 4.4. Scalability

As is stated in Section 2 of this research paper, a SDN infrastructure abstracts the control plane from deployed network devices via southbound interfaces. This control plane abstraction is managed by a single SDN controller that can address scalability issues when the topology hosts several switches. Several works have proposed distributed controller architectures [14,44,45] to address scalability issues.

Normally, video streaming applications are geographically distributed applications due to vendor or proprietary demands among others. However, the monitoring tool proposed in this paper has been designed for a generic network with a single SDN controller. Then, in order to address the scalability issue, the use of multiple SDN controllers for complex or bigger topologies adding a synchronization state protocol for the proposed algorithm should not be complicated.

## 5. Simulations and Results

**Figure 2:**
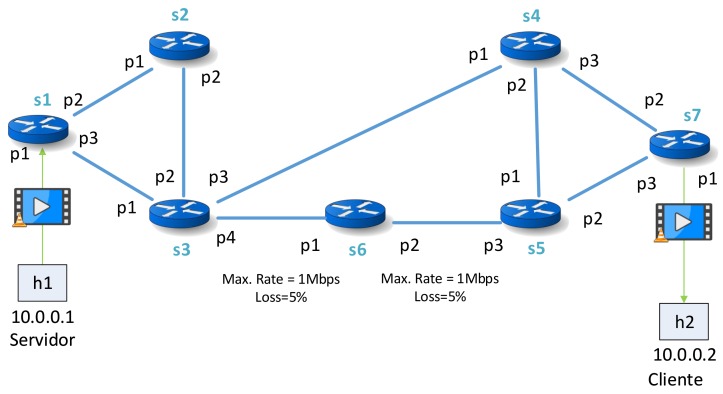
Topology Tested.

In this section, we test the feasibility of the Clustering enhanced technique with respect to the Flow-Conservation Algorithms. The simulations are tested using the topology described in Figure 2 which is composed of 7 OF-Switches ($s_{1},s_{2},s_{3},s_{4},s_{5},s_{6}$ and $s_{7}$) and 2 host ($h_{1}$ and $h_{2}$) connected to $s_{1}$ and $s_{7}$ respectively. The links [switch: $s_{3}$-port: $p_{4}$, switch: $s_{6}$-port: $p_{1}$] and [switch: $s_{6}$-port: $p_{2}$, switch: $s_{5}$-port: $p_{3}$] are configured with values of maximal data rate (1 Mbps of bandwidth) and loss percentage (5% of the package will be lost).

All simulations were run in a network simulator called Mininet v2.1.0 [46] using python scripts, which contains the topology to examine, as previously described. Mininet is able to create with few steps custom topologies to be simulated in a computer. The testing set was executed in a virtual machine within a ThinkServer model TD350 that is composed of an Intel Xeon Series E5-2600 v3 with 12 cores, 64 GB DDR4 RAM and Ubuntu 14.04 as Operative System. Inside the server is the VM, which is comprised of a Linux (Ubuntu v. 13.04) guest Operative System (64 bits, 8 GB RAM, 2 CPUs, 8 GB HD). Both the clustering and flow-conservation monitoring modules are implemented using the java-based Floodlight controller [13] inside such a VM.

A simulation consists of a video streaming sent from host 1 to host 2 using VLC video server and RTPUDP as streaming protocol at the same time that the monitoring module is running. Therefore, the main task of the monitoring module is to measure the statistics of network switches (both the links where the video is being sent and the rest of them). We use a set of videos for the video streaming where their features are gathered in Table 2 while the monitoring time (tmon) is 200 ms. The videos used in the videostreaming are “Highway_cif” [47], “Akiyo_cif” [48], “Bridge-far_cif” [49] and “Clarie_cif” [50]. The connection between the client host and server host is a fixed path composed of [switch: $s_{1}$-port: $p_{1}$, switch: $s_{1}$-port: $p_{3}$, switch: $s_{3}$-port: $p_{1}$, switch: $s_{3}$-port: $p_{4}$, switch: $s_{6}$-port: $p_{1}$, switch: $s_{6}$-port: $p_{2}$, switch: $s_{5}$-port: $p_{3}$, switch: $s_{5}$-port: $p_{2}$, switch: $s_{7}$-port: $p_{3}$, switch: $s_{7}$-port: $p_{1}$].

The testing set is divided into two study cases: Study Case A and Study Case B. The first group, Study Case A is comprised of a proof of concept where the differences between the clustering and flow conservation enhanced algorithms are manifested in the video streaming of “Highway_cif” [47]. Then, this study case is repeated twice in the same conditions changing the monitoring method. In the first one, the clustering enhancement proposed in this paper is used, and in the other, the method proposed in [43] is applied, which reduces the number of queries of 2-grade switches of the topology. Moreover, for each monitoring method, the test is repeated twice more in order to separate the values of each metric (data rate and error rate). Finally, the results are compared with these two enhanced modules to determine which method is efficient in terms of load and accuracy. The results from these tests are divided into three metrics: the number of requests to get the status of each switch, the data rate and error rate of the switches that compose the streaming path.

On the other hand, the Study Case B is focused on the applicability of the clustering algorithm in other videos (“Akiyo_cif” [48], “Bridge-far_cif” [49] and “Clarie_cif” [50]). The aim of this study case is to check the feasibility of our enhancement with different types of video in terms of frames, duration and bitrate and also different data rate patterns. Following the same procedure as the Study Case A, the test is repeated twice for each video to obtain its data rate.

The parameters for both Study Cases are: links [switch: $s_{3}$-port: $p_{4}$, switch: $s_{6}$-port: $p_{1}$] (hereafter called Link A) and [switch: $s_{6}$-port: $p_{2}$, switch: $s_{5}$-port: $p_{3}$] (hereafter called Link B) are set with a Bandwidth of 1 Mbps and a loss percentage of 5% respect the packets that are sent through these links.

### 5.1. Study Case A

Regarding the data rate simulations that we have performed, the number of monitoring queries in the non-enhanced method during simulation (80 s) is 2079 requests while in the enhanced algorithm is 903. The difference of 1176 queries shows a reduction ratio of 57% of requests with respect to a non-enhanced monitoring simulation as is gathered in Table 3.

**Figure 3:**
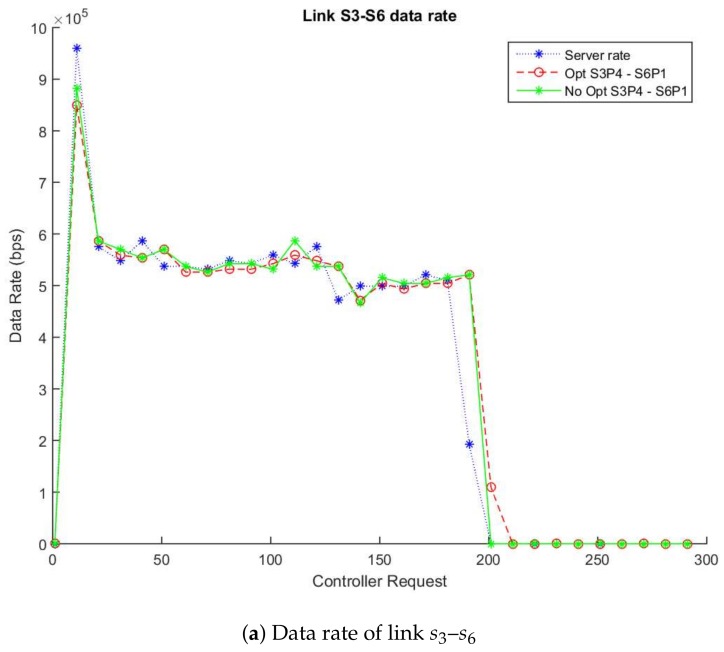

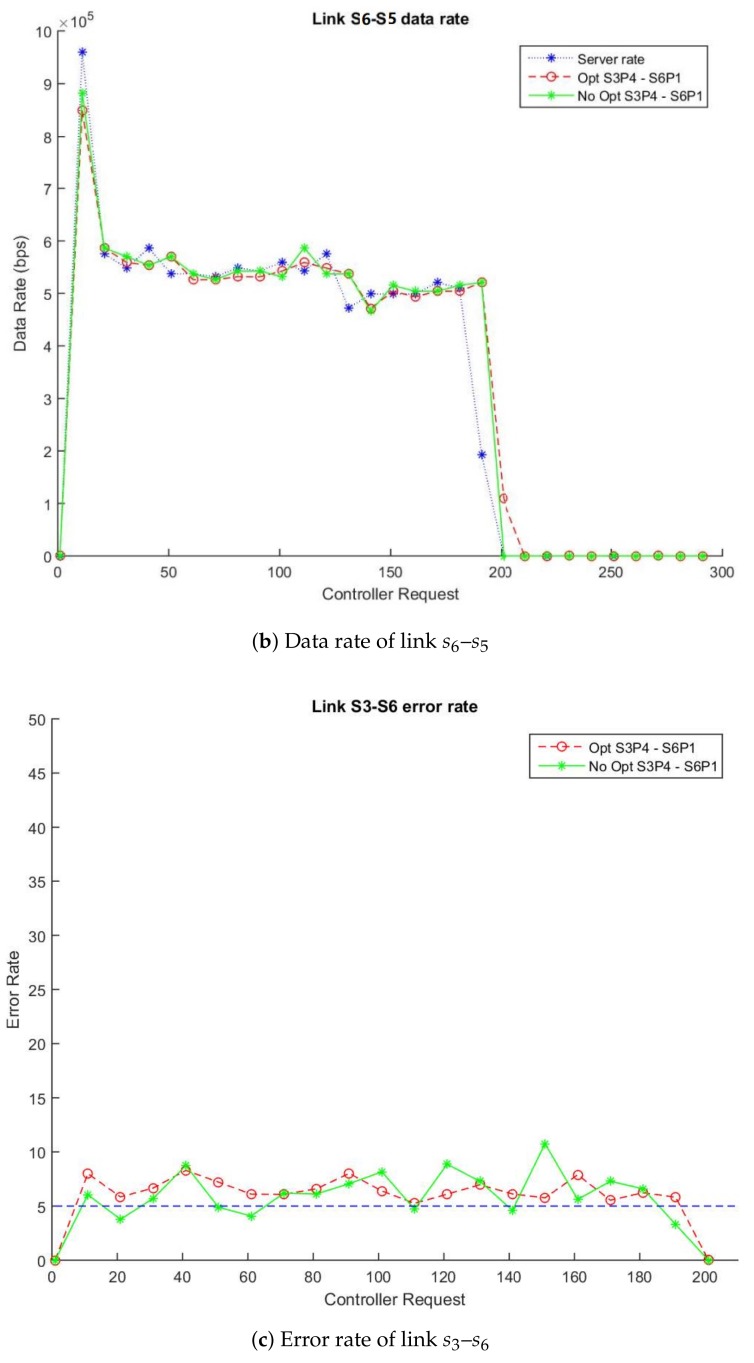

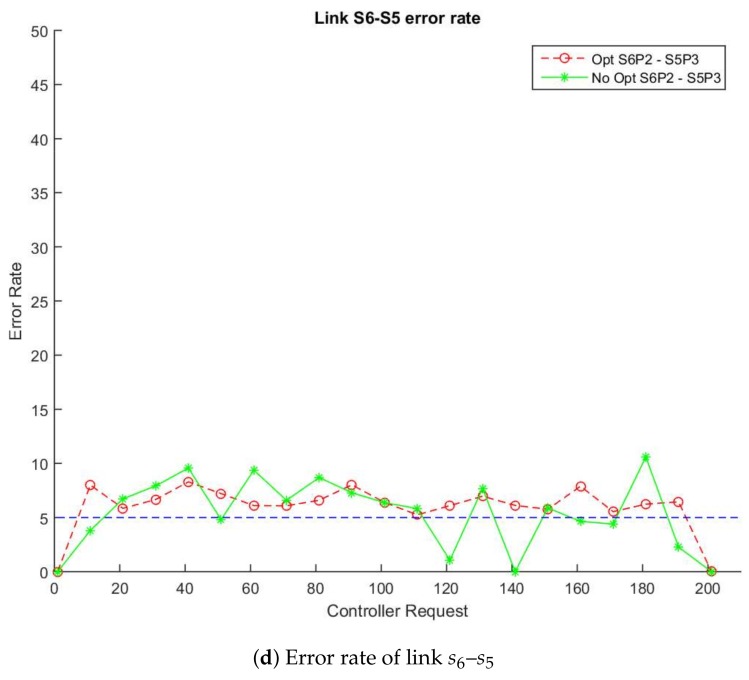
Enhanced vs. Non-Enhanced Data and Error Rate of Highway_cif.

For its part, Figure 3a,b show the traffic flow between two simulations in which one applies the enhancement and the other does not use it. Figure 3a describes the data flow (in bps) through the links $s_{3}$-$s_{6}$ (switch: $s_{3}$-port: $p_{4}$, switch: $s_{6}$-port: $p_{1}$) and Figure 3b the links between $s_{6}$ and $s_{5}$ (switch: $s_{6}$-port: $p_{2}$, switch: $s_{5}$-port: $p_{3}$). The pointed lines (blue lines) show the data rate that the server is sending the video while the solid lines (green lines) show the data rate obtained using the non-enhanced method and the dotted lines (red lines) display data rate with the enhanced algorithm. As expected, both links experiment with an increase of data rate due to the transmission of the video between $h_{1}$ and $h_{2}$. As soon as the transmission finishes (around 200 requests), the data rate decreases demonstrating the efficiency of the algorithm to detect changes in the network transmission. Similarly, the average difference between the links $s_{3}$–$s_{6}$ with/without the enhancement is 45.49 Kbps and 54.33 Kbps in the link $s_{6}$-$s_{5}$ as Table 3 describes. These results confirm that the enhancement maintains good levels of accuracy and reduces the number of requests in the data plane.

In the same way as the data rate metric, Figure 3c,d show the error rate in the traffic data over the streaming path. Figure 3c describes the error rate in the data flow (in bps) through the links $s_{3}$–$s_{6}$ (switch: $s_{3}$-port: $p_{4}$, switch: $s_{6}$-port: $p_{1}$) and Figure 3d between link $s_{6}$–$s_{5}$ (switch: $s_{6}$-port: $p_{2}$, switch: $s_{5}$-port: $p_{3}$). The solid lines (green lines) show the error rate obtained using the non-enhanced method, the dotted lines (red lines) display error rate with the enhanced algorithm and the dotted continue line (blue lines) shows the netem percentage configured in Mininet (5% packet loss). As expected, both links are able to detect the loss of information between the links due to the features of these links.

Therefore, based on our algorithm design, we can estimate approximately that a 57% message reduction is achieved in a topology similar to the experiment. This reduction belongs to the proportion between the accounted statistics messages in the links link $s_{3}$–$s_{6}$ and $s_{6}$–$s_{5}$ from the enhanced and non-enhanced simulations.

### 5.2. Study Case B

The average number of monitoring queries in the non-enhanced method during simulation is 2089 requests while in the enhanced algorithm the average value is 901. Then, the fact that the average difference is around 1186 queries shows a reduction ratio of 57% of requests with respect to a non-enhanced monitoring simulation as is stated in Table 4, Table 5 and Table 6.

**Figure 4:**
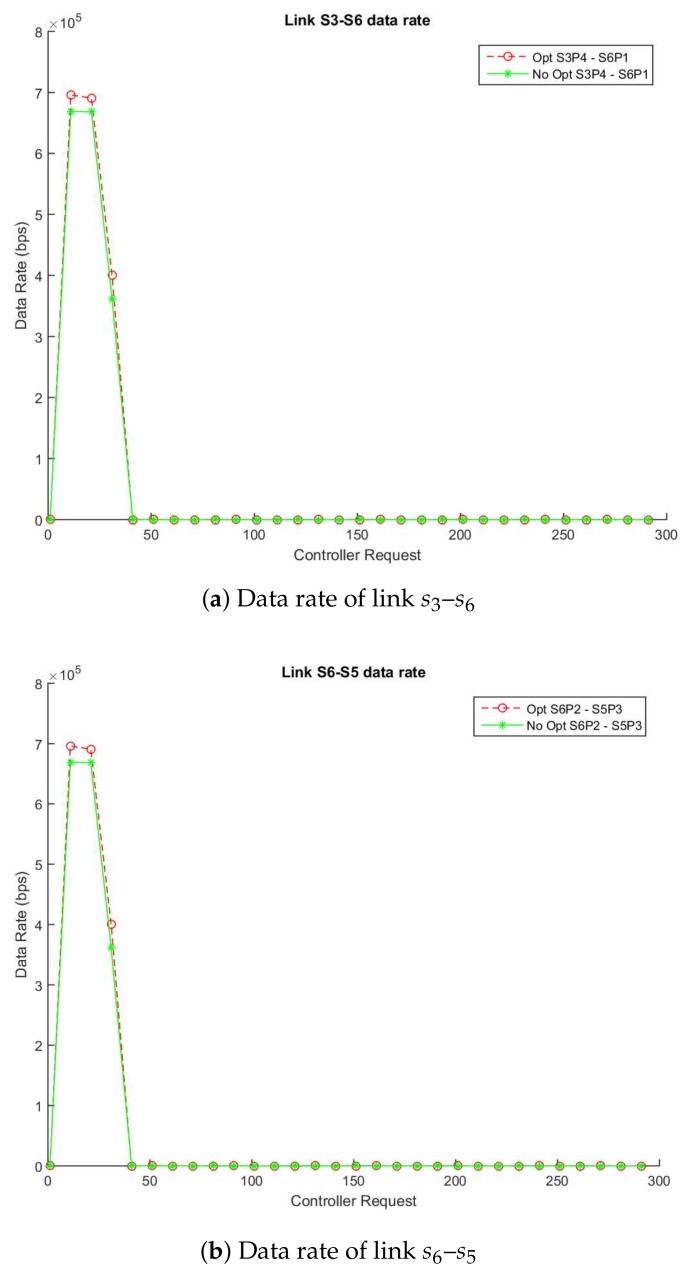
Enhanced vs. Non-Enhanced Data and Error Rate of Akiyo_cif.

**Figure 5:**
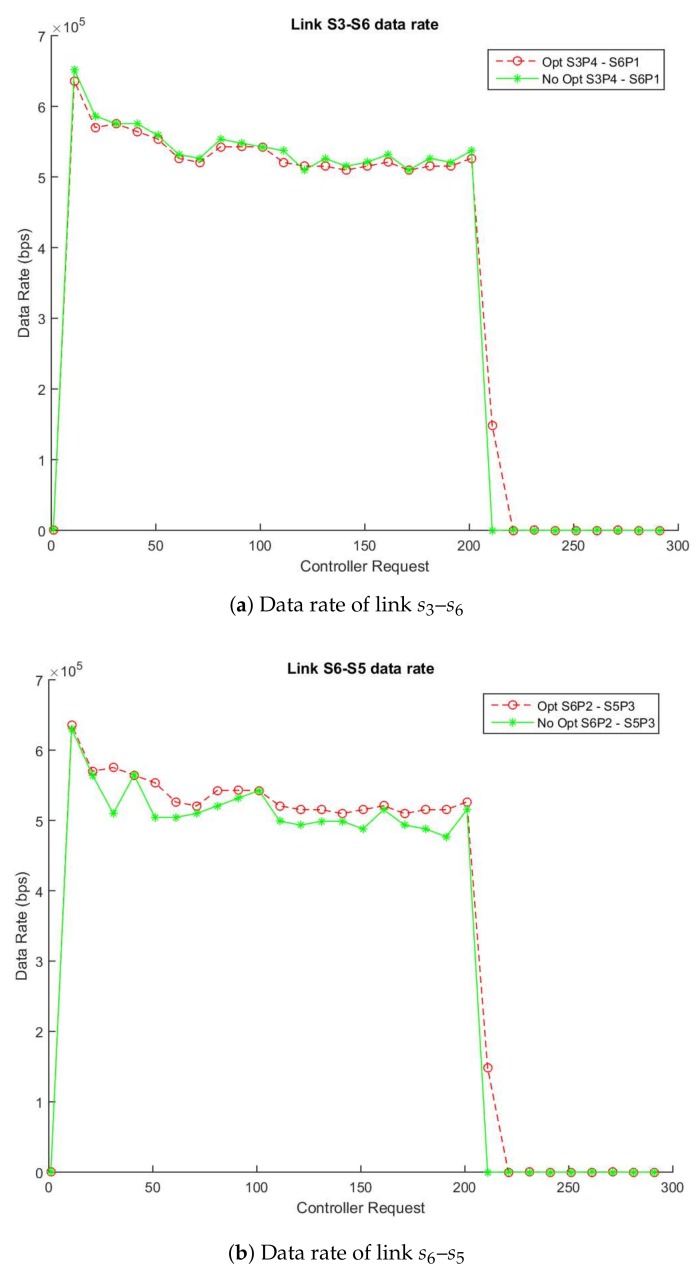
Enhanced vs. Non-Enhanced Data and Error Rate of Bridge-far_cif.

**Figure 6:**
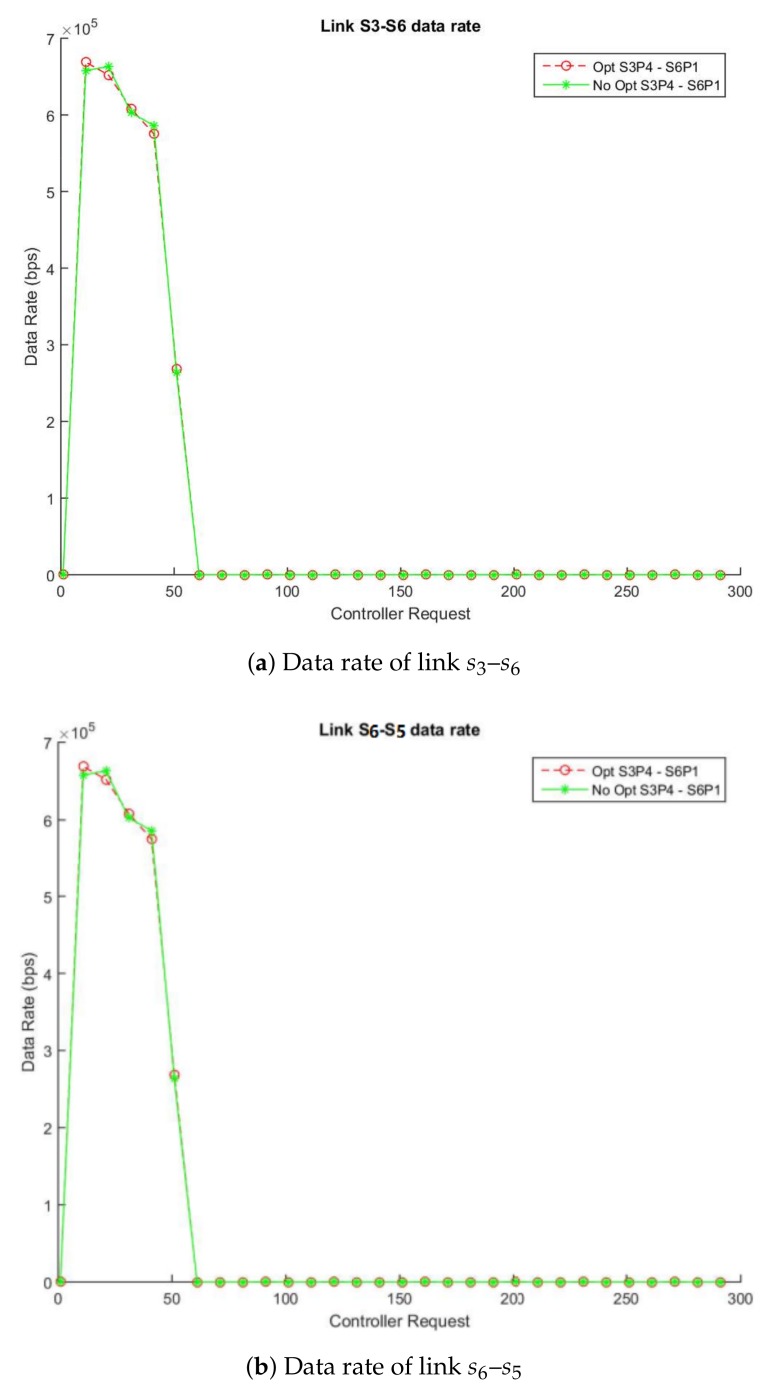
Enhanced vs. Non-Enhanced Data and Error Rate of Clarie_cif.

In the same way as the Study Case A, Figure 4a,b show the traffic flow in the video called “Akiyo_cif” [48], Figure 5a,b show the traffic flow in the video called “Bridge-far” [49] and finally, Figure 6a,b show the traffic flow in the video called “Claire” [50] between two simulations in which one applies the enhancement respect to another that does not use it. Figure 4a, Figure 5a and Figure 6a describe the data flow (in bps) through the links $s_{3}$–$s_{4}$ (switch: $s_{3}$-port: $p_{2}$, switch: $s_{4}$-port: $p_{1}$) and Figure 4b, Figure 5b and Figure 6b the links between $s_{4}$ and $s_{9}$ ((switch: $s_{4}$-port: $p_{2}$, switch: $s_{9}$-port: $p_{2}$). The solid lines show the data rate obtained using the non-enhanced method and the dotted lines display data rate with the enhanced algorithm. As expected, our enhanced method is able to monitor every type of video (no matter the traffic pattern) obtaining almost the same rate with very small differences and reducing to 57% the number of monitoring queries as is stated in Table 4, Table 5 and Table 6.

Based on our algorithm design with the videos used in the Study Case B simulations, we can estimate that approximately a 57% message reduction is achieved in a topology similar to the experiment. This reduction belongs to the proportion between the accounted statistics messages in the links link $s_{3}$–$s_{6}$ and $s_{6}$–$s_{5}$ from the enhanced and non-enhanced simulations.

To finish with the monitoring enhancement of our research work, both study cases show a reduction in the network traffic flow to keep free the SDN controller for other management actions.

## 6. Conclusions and Future Works

Users quantify their QoE in a video delivery being receptors of a non-stop streaming of instead of a good application quality. Hence, the goal for future networks is to dynamically adapt the QoE demands of users and applications that are using the networks.

SDN has changed the architecture of traditional networks separating the control and data planes removing its rigidity. In this way, SDN provides a unified view of the network providing scalability, flexibility and a centralized control.

A good monitoring tool is a crucial element in the network management since accurate and timely statistics on network devices are essential for management operations as load balancing, intrusion detection among others. Management applications may need to monitor network devices to obtain port traffic per unit time.

This paper presents an efficient SDN monitoring tool that reduces the number of monitoring requests clustering techniques in the devices of which the network is composed. This reduction in the queries to the network switches depends on the number of ports of the switches within a cluster.

To demonstrate the effectiveness of our monitoring tool, we have tested our enhanced technique using clustering algorithms to gather a statistics collection compared with not applying such enhancement in the same scenario. We have evaluated and compared the results in the same polling conditions. We have found that data rate values coming from the network switches are almost the same as the values coming from the data rate of the server that is providing the video streaming. Therefore, we have shown that our framework reduces the number of queries to the controller and the CPU load in the controller. Moreover, data rate and error rate values coming from the results of the simulations performed with the Mininet tool confirm the feasibility of such a framework due to the few differences between such values in enhanced and non-enhanced simulations.

As part of future work, we want to plan new strategies to reduce the monitoring queries as well as optimizations for measuring other variables such as delay, packet loss, jitter or packet duplication. Similarly, the use of active methods for monitoring SDN can be also taken into account to improve the quality of metrics. Moreover, there are new clustering algorithms such as density models (DBSCAN and OPTICS) or subspace models such as biclustering (also known as co-clustering).

Moreover, the study of scalability is a very interesting topic that will be take into account with the inclusion of more SDN controllers to interconnect complex or huge topologies with several servers and switches, hybrid topologies (both OpenFlow switches and nonOpenFlow switches) and topologies with different traffic flows (intermediate traffic flows).

## Figures and Tables

**Table 1:** Network Notation Model.

Symbol	Description
G=(S,L)	Network graph
*S*	Set of switches in the network Graph *G*
*L*	Set of links in the network Graph *G*
*C*	Set of clusters that contains switches
$s_{1},s_{2},\ldots ,s_{n}$	Generic switches in the network Graph *G*
$l_{1},l_{2},\ldots ,l_{n}$	Generic links in the network Graph *G*
$c_{0},c_{1},\ldots ,c_{nc-1}$	Generic clusters in the network Graph *G*
ns=|S|	Number of switches in *G*
nl=|L|	Number of links in *G*
nc=|C|	Number of clusters in *G*
arc(i,j)∈L	Link from source switch $s_{i}$ to destination switch $s_{j}$
$deg(s_{i})$	Number of links in switch $s_{i}$

**Table 2:** Features of videos used in the simulations.

Video	Format	Frames	Duration (s)	Size	Bitrate (kbits/s)
Highway_cif	MPEG 12	2000	80	2.5 MB	257.4
Akiyo_cif	MPEG 12	300	11.96	481.6 KB	329.8
Bridge-far_cif	MPEG 12	2101	84	2.95 MB	252.1
Clarie_cif	MPEG 12	494	19.72	712 KB	295.8

**Table 3:** Summary of Enhanced vs. Non-Enhanced Methods for Data Rate in Study Case A.

Method	Request Number	Improvement	Improvement (%)	Topology Link	Difference
Enhanced	903	1176	57%	Link $s_{3}$–$s_{6}$	45.49 Kbps
Non-Enhanced	2079			Link $s_{6}$–$s_{5}$	54.33 Kbps

**Table 4:** Summary of Enhanced vs. Non-Enhanced Methods for Data Rate in Akiyo video within Study Case B.

Method	Request Number	Improvement	Improvement (%)	Topology Link	Difference
Enhanced	900	1186	57%	Link $s_{3}$-$s_{6}$	3.42 Kbps
Non-Enhanced	2086			Link $s_{6}$-$s_{5}$	4.54 Kbps

**Table 5:** Summary of Enhanced vs. Non-Enhanced Methods for Data Rate in Bridge-far video within Study Case B.

Method	Request Number	Improvement	Improvement (%)	Topology Link	Difference
Enhanced	903	1183	57%	Link $s_{3}$-$s_{6}$	29.86 Kbps
Non-Enhanced	2086			Link $s_{6}$-$s_{5}$	39.8 Kbps

**Table 6:** Summary of Enhanced vs. Non-Enhanced Methods for Data Rate in Claire video within Study Case B.

Method	Request Number	Improvement	Improvement (%)	Topology Link	Difference
Enhanced	903	1190	57%	Link $s_{3}$-$s_{6}$	5.21 Kbps
Non-Enhanced	2093			Link $s_{6}$-$s_{5}$	8.72 Kbps

## Abbreviations

The following abbreviations are used in this manuscript:

API	Application Programming Interface
CDN	Content Delivery Network
DDoS	Distributed Denial of Service
HD	High Definition
HQ	High Quality
NETCONF	Network Configuration Protocol
NOS	Network Operative System
OF	OpenFlow
ONF	Open Networking Foundation
OS	Operative System
QoE	Quality of Experience
QoS	Quality of Service
SDN	Software Defined Network
SNMP	Simple Network Management Protocol
TLS	Transport Layer Security
VLAN	Virtual Local Area Networks
